# Interiors of small bodies and moons

**DOI:** 10.1038/s41467-020-15458-8

**Published:** 2020-03-26

**Authors:** Erik Asphaug

**Affiliations:** Lunar and Planetary Laboratory University of Arizona Tucson, Tucson, AZ 85721 USA

**Keywords:** Astronomy and astrophysics, Planetary science

## Abstract

Asteroids, comets and moons are leftovers of planet formation. Studying them and their samples, including meteorites, can help us to learn how the Earth was made and acquired the ingredients for life, to obtain practical information for deflecting near-Earth objects (NEOs), and to access resources that would enable space habitats and voyages. Answers are hidden beneath their complex and evolving exteriors.

## A book by its cover

**Fig. 1:**
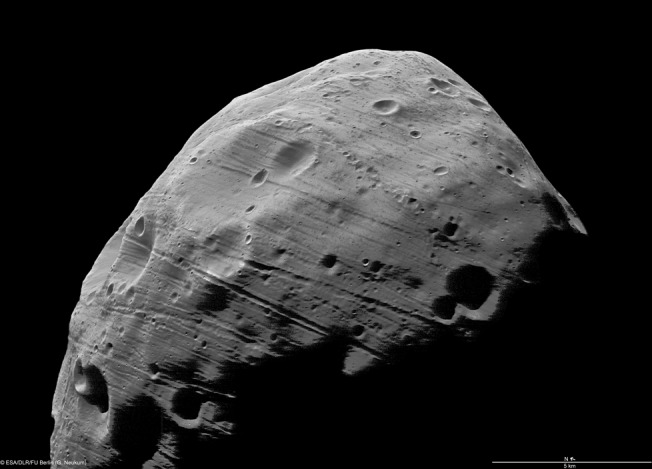
The Martian satellite Phobos was the first small body imaged in detail. It orbits so close to Mars that without friction or strength it would fall apart due to tides. It is spiraling in and expected to come apart in tens of millions of years, forming a debris ring and causing an intense planetary equatorial bombardment. Shown is one of the highest resolution images yet obtained (scale bar 5 km; 3.7 m/pixel), acquired by Mars Express whose polar orbit sometimes crosses that of Phobos. The network of parallel grooves correlate with the direction of growing tidal strain, and may be a precursor to global failure and a hint at what’s inside (ESA/DLR/FU Berlin).

In the 1970s, Mariner 9 and Viking 1 obtained the first geologic images of small planetary bodies^1^, Phobos and Deimos, the moons of Mars (Fig. 1). Since then spacecraft have visited an astonishing diversity of battered relics, providing images of their geology and data about their compositions, and sometimes interacting with their surfaces by collecting samples and making exploratory craters. Astronomers have characterized spectral, thermal and rotational properties of thousands of them, and discovered that many comets and asteroids are binary or triple-systems. Radio telescopes, larger than many of the NEOs they track, measure their orbits, rotations, surface roughness, and for the ones that come close, their detailed shapes^2^.

Yet beneath these observations their interiors harbor secrets^3^. The most obvious clue is mass, measured by tracking a natural moon or orbiting spacecraft. The bulk densities of small bodies are quite low compared to the rocky or icy materials they are made of, implying they are porous. While this is consistent with the popular idea that they are loosely packed rubble piles, inspections by spacecraft show clear evidence of strength, at least on those that are larger than a few kilometers. Hence the also-popular idea that small bodies are monoliths riddled with fractures. Can both ideas be right?

## Interiors of comets

Comets, once captured by the inner solar system, are not long for this world. They vanish by sublimation of their ices with every perihelion. Comet 9P/Tempel 1 experienced decameters of scarp retreat between 2005 and 2011, and 67P/Churyumov-Gerasimenko lost hundreds of millions of tons of gas and dust between 2014 and 2016. Comets are also destroyed by close encounters with planets and the Sun, the best-documented example being Shoemaker-Levy 9 (SL9) that was pulled apart by Jupiter’s gravity field into a chain of 20 nuclei in 1992. It responded to the tidal stress as a cohesionless granular solid with bulk density 0.5 g/cm^3^—one might think, an icy rubble pile^4^.

**Fig. 2:**
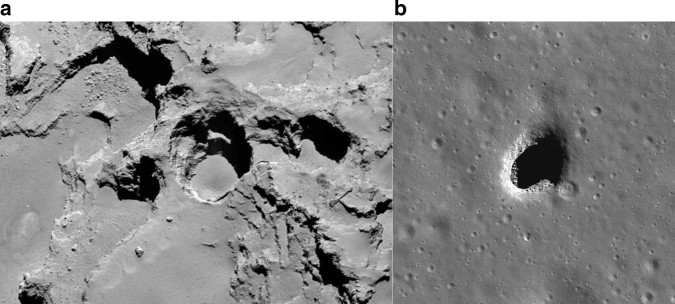
Volatile-rich and igneous bodies have holes to their interiors that we can explore. **a** Sinkholes in comet 67P/Churyumov-Gerasimenko, where material has collapsed almost 200 m into voids associated with cometary activity (ESA/Rosetta/MPS). **b** One of the skylights discovered on the nearside volcanic plains of the Moon. This 130-m cave in Mare Ingenii probably formed when the roof of an underground lava tube collapsed (NASA/LROC/ASU).

The shape of 67P resembles two spheroids in contact, and its bulk density is the same as SL9, so it might too be a rubble pile. Yet the breathtaking landscapes imaged by the Rosetta mission feature competent structures^5^ at regional to global scales, deep chasms and pits (Fig. 2a) and a kilometer-high cliff riddled with fractures. The Philae probe banged onto a hard surface. On its way down, Philae looped behind the nucleus, recording a 90-MHz radio signal from Rosetta. The experiment ended early but was filled with surprises. The complex surface and kilometers of nucleus material proved radar-transparent. The interior appeared homogeneous at decameter scales, despite the exterior expressions of structure. And instead of being made of mostly ice, it seems to be an icy mix of mostly silicates and organics^6^. If so, this would not only change how we think about the inventory of planet-forming materials, but would require an interior structure dominated by voids—porosity of at least 80%—yet with the ability to support towering cliffs and fractured structures.

We’d like to sample what’s inside, bringing back unprocessed material from before the birth of planets. To do that we have to get through the meters-thick lag of silicates, organics and reprocessed ices left behind by sublimation and altered by radiation, at least on comets such as 9P and 67P (P for periodic) that are accessible to missions. We might have to excavate or sample a newly exposed surface in a hazardous location. It might make initial sense to follow up on Rosetta’s interrogation and have an orbiter acquire thousands of radar echoes from every orientation, at tens of meters range-resolution, as the nucleus rotates like a patient on the table. Wave-based 3D image processing^7^ would resolve the dielectric constant (related to composition and porosity) and produce a detailed 3D contrast image that would show how the nucleus accreted (i.e., from what size progenitors and at what velocity), whether it’s a fragment of a larger body or has buried craters, how the interior relates to surface features and cometary activity, and where to sample truly primitive materials.

## Interiors of asteroids

Unlike comets, whose volatile-rich fragments vaporize on impact with the atmosphere, we have thousands of samples of rocky asteroid interiors. Meteorites derive mostly from disrupted NEOs that derive from disrupted progenitors; it’s putting them in context and accounting for the biases that’s the challenge. Some meteorites represent interiors of large differentiated asteroids, their mineralogy indicating their depth of origin: iron meteorites from cores, eucrites from crusts, and so on. When NASA’s mission to Psyche arrives in 2026, it will study a 250-km relic once thought to be the metallic interior of a disrupted Vesta-sized progenitor. Its story is more complex^8^. Asteroids larger than Psyche are too big to have ever been disrupted, according to theory, except for SL9- like grazing encounters with terrestrial planets during their formation. Smaller asteroids are the products of billions of years of collisional grinding. Psyche is inbetween.

A rubble pile asteroid grazing Earth’s tidal field would come apart like SL9, although an impact with Earth is more likely. While we haven’t seen either happen in modern times, there is strong evidence that asteroids, like comets, have rubble pile interiors. The first NEO seen up close, the ~300-m peanut-shaped Itokawa^9^, has a gravity field 1/100,000 that of Earth. Theory indicated it would be monolithic, unable to hold onto loose pieces, yet images showed it to be piled full of rocks, with a sea of regolith along a gravitational equipotential^10^. Ryugu and Bennu, about twice as big, are also piled with rocks, and have top-shapes and densities (1.2 g/cm^3^) consistent with rotating, gravitating rubble. Indeed, most asteroids and cometary nuclei have near-equilibrium shapes. Other evidence comes from the fact that small NEOs can get spun to fast rotation by sunlight, the YORP effect. Fast rotation causes a rubble pile to deform^11^ and even mutate into binaries or separated pairs, responding as granular solids^12^, in agreement with the observed population.

Asteroids can also be explored by radar, but their interiors are more scattering and opaque. Seismology is another way of probing deep inside, and provides mechanical knowledge vital to hazardous asteroid deflection and advanced operations like mining. Seismology needs a source, an artificial explosion or impact, or native activity like tidal creaking in Phobos or particle ejection events like on Bennu^13^, or cometary activity. The receiver must be embedded or anchored in microgravity, and that requires having operational knowledge of the regolith already. A similar bootstrapping challenge faces drilling and penetrometry; presently these approaches must be ready for resistant boulders and airless silt. One exciting possibility is doing seismology from orbit, using an industry technique, laser Doppler vibrometry, to turn partially buried boulders into seismic stations to resolve internal structure^14^.

We can also use impacts and explosions to punch craters, as done by Deep Impact and Hayabusa 2 which excavated the near-surfaces of Tempel 1 and Ryugu. When NASA’s DART mission tests asteroid deflection by crashing into the 140-m moon of Didymos in 2022, it might cause global damage and expose a deep-probing crater. An Italian cubesat will separate before the impact and fly through the event. The European Space Agency's Hera mission will visit the system a few years later and obtain a comprehensive view, deploying a lander, and performing a radar experiment analogous to Rosetta’s.

## Phobos and beyond

One of the most striking features on Phobos is its network of parallel grooves, 10–100 meters wide (Fig. 1), that appear preferentially aligned^15^ with the increasing strain of tidal deformation. At meters-resolution they look like cracks in rock, but another explanation is that they are fissures in dusty regolith, the way that cracks appear in the powdery surface of the Moon. The gravity being 300 times weaker, the spacing of fissures suggests a regolith cohesion of about 1 kPa, comparable to lunar regolith. If so, what does this imply for the interior? And how did these moons get emplaced around Mars in the first place? We’ll know much more when the Japanese Aerospace Exploration Agency's MMX mission goes into orbit around 2025, deploys its landers, and brings back samples.

Looking deeper, bodies up to the size of Phobos have central pressures of less than 1 bar, so if they are made of rubble or have fissures, then a burrowing robot could explore the deep interior, assuming engineers can figure out power, communication and thermal problems. Using more traditional technologies, robots can thread their way through caverns and interconnected voids^16^, and even cometary vents, the way they will investigate skylights into lava tubes on the nearside of the Moon (Fig. 2b). Networked teams could explore the deepest reaches, discovering geological deposits, paving the way for off-world habitats, and advancing the quest for subterranean life on Europa and Mars.

In the process we’ll learn how planets are made. Expansion on these ideas are found in ref. ^17^.
